# Supplementary material for: Nationwide Laboratory Surveillance of Progressive Multifocal Leukoencephalopathy in Japan: Fiscal Years 2011–2020
Source: Viruses. 2023 Apr 14;15(4):968. doi: 10.3390/v15040968 (PMC10144269; doi:10.3390/v15040968)
Supplement: Supplementary file 1 [file viruses-15-00968-s001.zip › Supplementary_Figure_S1_Nakamichi_et_al.pdf]

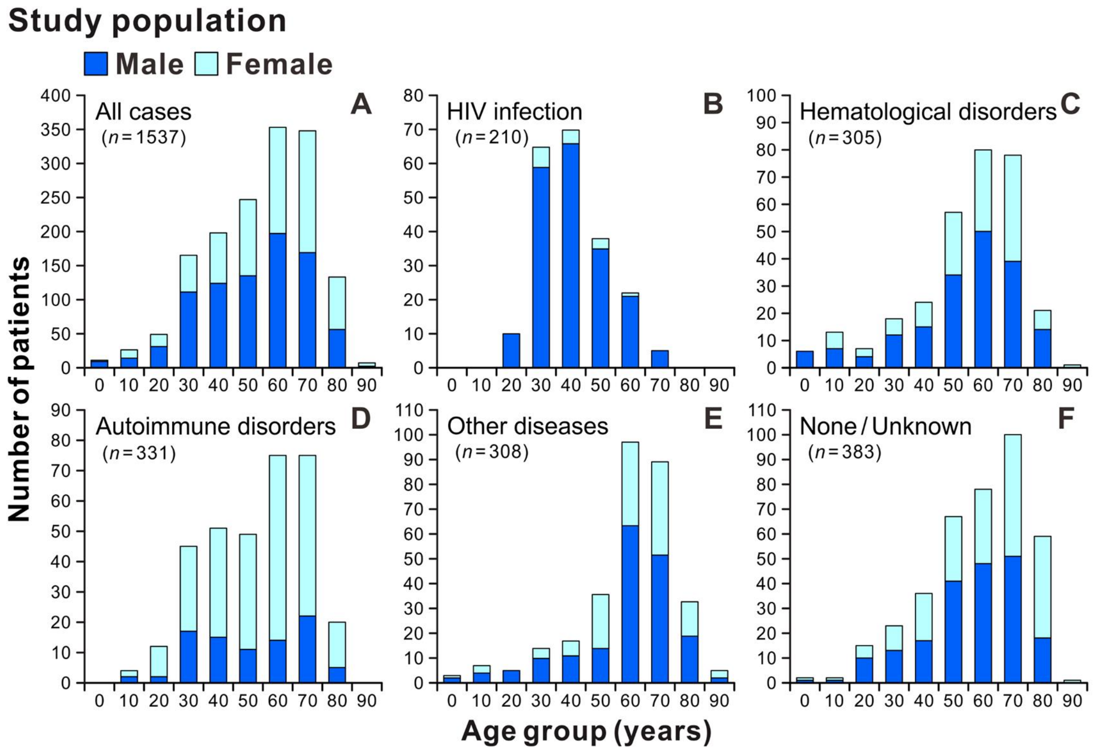

Supplementary Figure S1. Age and sex distribution patterns of the study population. Individuals who underwent CSF-JCV testing during the study period (A) were divided into five groups based on underlying conditions: HIV infection (B), hematologic disorders (C), autoimmune disorders (D), other diseases (E), and none/unknown (F). The "other diseases" group includes individuals with a history of organ transplantation, solid tumors, or comorbidities of multiple underlying diseases. The vertical axes show the number of patients, and the numbers below the bars indicate the age groups by decade.
