# Supplementary material for: Nationwide Laboratory Surveillance of Progressive Multifocal Leukoencephalopathy in Japan: Fiscal Years 2011–2020
Source: Viruses. 2023 Apr 14;15(4):968. doi: 10.3390/v15040968 (PMC10144269; doi:10.3390/v15040968)
Supplement: Supplementary file 1 [file viruses-15-00968-s001.zip › Supplementary_Table_S1_Nakamichi_et_al.pdf]

Supplementary Table S1. Underlying disease categories and detection rates in areas with high proportions of patients with JCV-positive CSF

Category	No. (%) of JCV-positive cases [a]				
	Clusters 1–2		Clusters 3–5		*P*-value [b]
HIV infection	12/25	(48.0)	45/185	(24.3)	0.017 *
Hematological disorders	29/70	(41.4)	49/235	(20.9)	<0.001 *
Autoimmune disorders	16/46	(34.8)	59/285	(20.7)	0.055
Organ transplantation	0/5	(0)	12/42	(28.6)	0.308
Solid organ tumors	2/10	(20.0)	6/80	(7.5)	0.216
Other diseases	12/27	(44.4)	28/144	(19.4)	0.011 *
None/unknown	3/33	(9.1)	15/350	(4.3)	0.195
Total	74/216	(34.3)	214/1321	(16.2)	<0.001 *

Abbreviations: CSF, cerebrospinal fluid; HIV, human immunodeficiency virus; JCV, JC virus.

[a] Number of CSF JCV-positive cases and positive rates (%) in the clusters shown in Figure 2D.

[b] The positive rates of CSF JCV in clusters 1–2 and 3–5 are statistically analyzed using Fisher's exact test. Significant differences are indicated by asterisks ($P < 0.05$).
